# Royal United Hospital, Bath

**Published:** 1904-08-13

**Authors:** 


### EDITOR'S LETTER-BOX.

### ROYAL UNITED HOSPITAL, BATH.

Mr. J. M. Sheppard, Secretary of the Royal United Hospital, Bath, writes: In your issue of July 30th I notice you have given us a paragraph under "Glances at the Hospitals," part of which is extremely misleading to your readers. You say "the income amounted to £5,774." (This included legacies (£717), which cannot be looked upon as "income.") Then you say "the expenditure was £10,071." This is altogether erroneous. The balance due to the treasurers on January 1st, 1902, was £3,370 3s. 7d. The actual expenditure was £6,701, and the balance due to the treasurers on December 31st had increased to £4,297.

